# Production of organic flavor compounds by dominant lactic acid bacteria and yeasts from *Obushera,* a traditional sorghum malt fermented beverage

**DOI:** 10.1002/fsn3.450

**Published:** 2016-12-05

**Authors:** Ivan M. Mukisa, Yusuf B. Byaruhanga, Charles M. B. K. Muyanja, Thor Langsrud, Judith A. Narvhus

**Affiliations:** ^1^ Department of ChemistryBiotechnology and Food Science (IKBM)Norwegian University of Life Sciences (UMB)ÅsNorway; ^2^ Department of Food Technology and NutritionMakerere UniversityKampalaUganda

**Keywords:** Cereal fermentation, lactic acid bacteria, *Obushera*, sorghum, starter cultures, Yeasts

## Abstract

Single and mixed starter cultures of lactic acid bacteria (LAB): *Weissella confusa* MNC20, *Lactobacillus plantarum* MNC21, *Lactococcus lactis* MNC24 and *Lactobacillus fermentum* MNC34 and yeasts: *Issatchenkia orientalis* MNC20Y and *Saccharomyces cerevisiae* MNC21Y were used to produce *Obushera*, a fermented sorghum beverage. Microbial counts, pH, sugars, organic acids, and volatile compounds in starter culture and spontaneous fermentations were monitored during 48 hrs. Maximum counts of LAB (8.4–9.4 log cfu g^−1^) and yeasts (7.5 ± 0.1 cfu g^−1^) starter cultures were attained in 6–48 hrs. *Weissella confusa*,* Lc. lactis*, and *Lb. fermentum* showed possible acid sensitivity while *I. orientalis* produced surface films. LAB starter cultures and their combinations with *S. cerevisiae* lowered pH from 5.83 to <4.5 (3.50–4.13) in a shorter time (12 hrs) than spontaneous fermentations (24 hrs). *Lactococcus lactis* and *W. confusa* metabolized glucose the fastest (*p* < .05) during the first 6 hrs. *Lactobacillus fermentum, Lb. plantarum*, and *S. cerevisiae* utilized glucose and maltose concurrently. *Lactobacillus plantarum* and *S. cerevisiae* additionally utilized fructose. *S. cerevisiae* metabolized sugars the fastest (*p* < .05) during the first 12–24 hrs. *Lactobacillus plantarum* and *W. confusa* produced the highest (*p* < .05) amounts of lactate (5.43 g kg^−1^) and diacetyl (9.5 mg kg^−1^), respectively. LAB also produced acetate, ethanol, acetaldehyde, acetone, and acetoin. Coculturing LAB with *S. cerevisiae* reduced (*p* < .05) lactate and diacetyl yield. Yeasts produced high amounts of acetaldehyde and methyl alcohols. *Issatchenkia orientalis* produced higher (*p* < .05) amounts of 2‐methy‐1‐propanol and 3‐methyl‐1‐butanol than *S. cerevisiae*. Combinations of LAB with *S. cerevisiae* produced a profile flavor compounds close to that of spontaneously fermented *Obushera*. These combinations can be adopted for controlled fermentation of *Obushera* and related fermented cereal products.

## 1 Introduction

Traditional fermented cereal products are important in nutrition as sources of carbohydrates, proteins, fiber, minerals, and vitamins (Nout, 2009). In different parts of Africa, the main cereals: maize, sorghum, and millet – are widely used to produce fermented nonalcoholic and alcoholic foods such as *Mageu* (Holzapfel & Taljaard, 2004), *Togwa* (Mugula, Narvhus, & Sørhaug, 2003), *Gowé* (Vieira‐Dalodé et al., 2007), *Poto poto* and *Degué* (Abriouel et al., 2006), and *Obushera* (Mukisa, Muyanja, Byaruhanga, Langsrud, & Narvhus, 2012) among others. There is great interest in industrializing traditional fermented products from different parts of the world, including Africa (Tamang & Kailasapathy, 2010).

Relying on natural fermentation is a major hindrance to large‐scale commercial processing of traditional fermented products mainly because natural fermentation is associated with variations in product quality and safety (Holzapfel, 2002). Starter cultures with desirable properties can be identified and applied to ensure reduction in processing time, consistent product quality, and safety (Holzapfel, 2002). However, selecting appropriate starter cultures requires understanding the microbial diversity of the products and the roles played by specific organisms.

Lactic acid bacteria (LAB) and yeasts are the major microbes involved in the fermentation of various traditional cereal products (Holzapfel, 2002; Nout, 2009). Culture‐ independent techniques revealed the predominance of LAB, including *Lactobacillus (Lb) plantarum, Lb. fermentum, Pediococcus (P). pentosaceus, Lb. delbrueckii, Lb. casei, Lb. curvatus, Lactococcus (Lc.) lactis, Weissella (W) confusa* and *W. cibaria*; and yeasts, including: *Saccharomyces cerevisiae*,* Issatchenkia orientalis, Candida* spp, and *Pichia* spp (Ampe, ben Omar, Moizan, Wacher, & Guyot, 1999; Madoroba et al., 2011; Mukisa, Porcellato, et al., 2012; ben Omar & Ampe, 2000).

Some studies evaluating starter cultures in traditional cereal fermentations focused on LAB and mostly on their ability to rapidly acidify the products (Agarry, Nkama, & Akoma, 2010; Muyanja, Narvhus, & Langsrud, 2012; Sekwati‐Monang & Gänzle, 2011). Others evaluated combinations of LAB and yeasts (Halm, Osei‐Yaw, Hayford, Kpodo, & Amoa‐Awua, 1996; Mugula et al., 2003; Omemu, Oyewole, & Bankole, 2007; Orji, Mbata, Aniche, & Ahonkhai, 2003). Products fermented with both LAB and yeasts have a flavor profile (Mugula et al., 2003) and organoleptic properties comparable to those of naturally fermented products (Halm et al., 1996; Masha, Ipsen, Petersen, & Jakobsen, 1998; Orji et al., 2003).

The flavor profile of cereal fermented products is composed of sugars, organic acids, aldehydes, ketones, alcohols, and esters (Mugula et al., 2003; Mukisa et al., 2012; Muyanja, Narvhus, & Langsrud, 2012). In sourdough fermentation, LAB are mainly responsible for acidification while yeasts produce ethanol, methyl alcohols, methyl aldehydes, and acetaldehyde among other volatiles (Gobbetti et al., 1995; Hansen & Hansen, 1996). The contribution of LAB toward production of volatile organic compounds in traditional African cereal fermented products has not been given much attention. Although the interaction between LAB and yeasts is known to enhance growth of either group of microbes (Mugula et al., 2003; Omemu et al., 2007), the effect of this interaction on flavor production has not been reported. Furthermore, differences in production of volatile organic compounds by the yeasts have also not been evaluated.

This study evaluated the individual and interactive effects of the dominant yeasts: *S. cerevisiae* and *I. orientalis* and the different LAB (*Lb. plantarum, Lb. fermentum, W. confusa, Lc. lactis*) on the flavor profile of traditional fermented cereals. *Obushera,* of the *Obutoko* type, a popular Ugandan fermented beverage prepared from malted sorghum (Mukisa, 2012) was used as a model for comparison. Information generated from this study will enable the identification of potential starter cultures for traditional fermented cereal products.

## 2 Materials and Methods

### 2.1 Bacteria and yeast starter cultures

*Lactobacillus plantarum* MNC 21, *W. confusa* MNC 20, *Lc. lactis* MNC 24, *Lb. fermentum* MNC 34, *I. orientalis* MNC 20Y and *S. cerevisiae* MNC 21Y (Gene bank accession numbers: JF512470, JQ754455, JF512471, JQ754464, JQ754435, and JQ754436, respectively) were used in this study. These LAB and yeasts were previously isolated from *Obushera* and identified by sequencing 16S rRNA and the ITS1‐5.8S ‐ITS2 region of rRNA, respectively (Mukisa, Porcellato, et al., 2012). The above strains were selected for this study since they belong to the dominant genera and species involved in the fermentation of *Obushera* (Mukisa et al., 2012). The LAB and yeasts were initially used as single starter cultures and later as mixed starter cultures. Mixed starter cultures included a primarily homolactic combination of *Lb. plantarum* + *Lc. lactis*. Coculturing *Lc. lactis* with *Lb. plantarum* was previously observed to enhance acid production in a cereal fermentation (Mukisa, Byaruhanga, et al., 2012). This starter culture therefore acted as a basis for two heterolactic combinations: *Lb. plantarum* + *Lc. lactis* + *W. confusa* and *Lb. plantarum* + *Lc. lactis* + *Lb. fermentum*. The yeast strain *S. cerevisiae* was used in two LAB/yeast mixed starter cultures: *Lb. plantarum* + *Lc. lactis* + *S. cerevisiae* (with homolactic LAB) and *Lb. plantarum* + *W. confusa* + *S. cerevisiae* (with homolactic and heterolactic LAB)*. I. orientalis* was not used in the mixed starter cultures because it formed undesirable white films in the product.

Pure LAB and yeast isolates were maintained at −80°C in broth containing 15% glycerol (v/v): MRS (Merck KGaA, Darmstadt, Germany) and tryptone glucose yeast extract broth (Oxoid Ltd, Hampshire, UK), respectively. Starter cultures for inoculation were prepared by growing strains in 250 ml of respective broths at 30°C for 24 hrs. Cells were recovered by centrifugation (7,500 × g for 10 min at 4°C) and subsequently rinsed with sterile distilled water. The cell pellet was resuspended in 25 ml of Ringer's solution containing 15% glycerol and stored in aliquots at −80°C. The resulting suspension contained approximately 10^9^ cfu ml^−1^ (for LAB) or 10^7^ cfu ml^−1^ (for yeasts) determined by taking plate counts on de Man, Rogosa and Sharpe (MRS) agar and Rose Bengal Chloramphenicol agar (RBCA), respectively (Merck KGaA, Darmstadt, Germany).

### 2.2 Preparation of *Obushera* samples

*Obushera* of the *Obutoko* type (from malted sorghum) was prepared using flour from red sorghum (*Sorghum bicolor* (L.) Moench) of the Sekedo variety. One batch of sorghum malt was prepared as described earlier (Mukisa, Muyanja, Byaruhanga, Schüller et al., 2012). The formulation described by Mukisa et al. (2010) was used to prepare *Obushera*. Briefly, slurries were made by mixing 50 g of sorghum malt in 400 ml of sterile distilled water held in 1 L glass jars. The slurries were heated in a water bath to 90°C, held for 10 min and then cooled to 30°C prior to inoculation. The slurries were then inoculated with single or mixed starter cultures resulting in initial cell concentrations of 6.6 ± 0.2 log cfu g^−1^ and 4.5 ± 0.3 log cfu g^−1^ for individual LAB and yeasts strains, respectively (Mukisa, 2012). Natural fermentation was initiated by adding 9 g of sorghum malt (2% malt) to the slurries (Mukisa et al., 2010). After inoculation, samples were homogenized for 1 min in an Omni mixer (Omni International, Waterbury, USA). Approximately 100 ml of sample was aseptically transferred into 200 ml sterile glass jars and incubated at 30°C. Samples were taken at *t* = 0, 6, 12, 24, and 48 hrs for the analysis of pH, titratable acidity, sugars, organic acids, volatile organic compounds, and microbial counts. Each sampling time was allocated a separate container. Three independent fermentations were made for each starter culture combination.

### 2.3 Microbial counts

LAB and yeast counts were determined by plating appropriate serial dilutions of *Obushera* in ¼ strength Ringer's solution on MRS agar and RBCA, respectively. LAB counts were taken after incubation at 30°C for 2–3 days while yeasts were counted after incubation at 25°C for 2–5 days.

### 2.4 Determination of pH, sugars, organic acids, and volatile organic compounds

The pH of *Obushera* was measured, using a pH meter (PHM61, Radiometer, Copenhagen, Denmark) equipped with a glass electrode (Type PHC2001‐8, Radiometer Analytical SAS, Villeurbanne Cedex, France). Concentrations of sugars and organic acids were determined with high performance liquid chromatography (HPLC) as previously described (Mukisa, 2012; Narvhus, Østeraas, Mutukumira, & Abrahamsen, 1998). Volatile organic compounds were analyzed by automatic headspace gas chromatography (HS‐GC), using the method described by Narvhus et al. (1998). The compounds analyzed constitute the main flavor compounds in traditional African cereal products (Mugula et al., 2003; Mukisa, 2012; Muyanja, Narvhus, & Langsrud, 2012).

### 2.5 Data analysis

Results were presented as arithmetic means ± standard deviations (Mean ± SD) of three independent fermentations. Data were subjected to one‐way analysis of variance (ANOVA) to test for significant differences at *p* = .05. Mean comparisons were made, using the Fisher's least significant difference (LSD) test to determine which means were significantly different.

## 3 Results

### 3.1 Growth of starter cultures in *Obushera*

**Figure 1:**
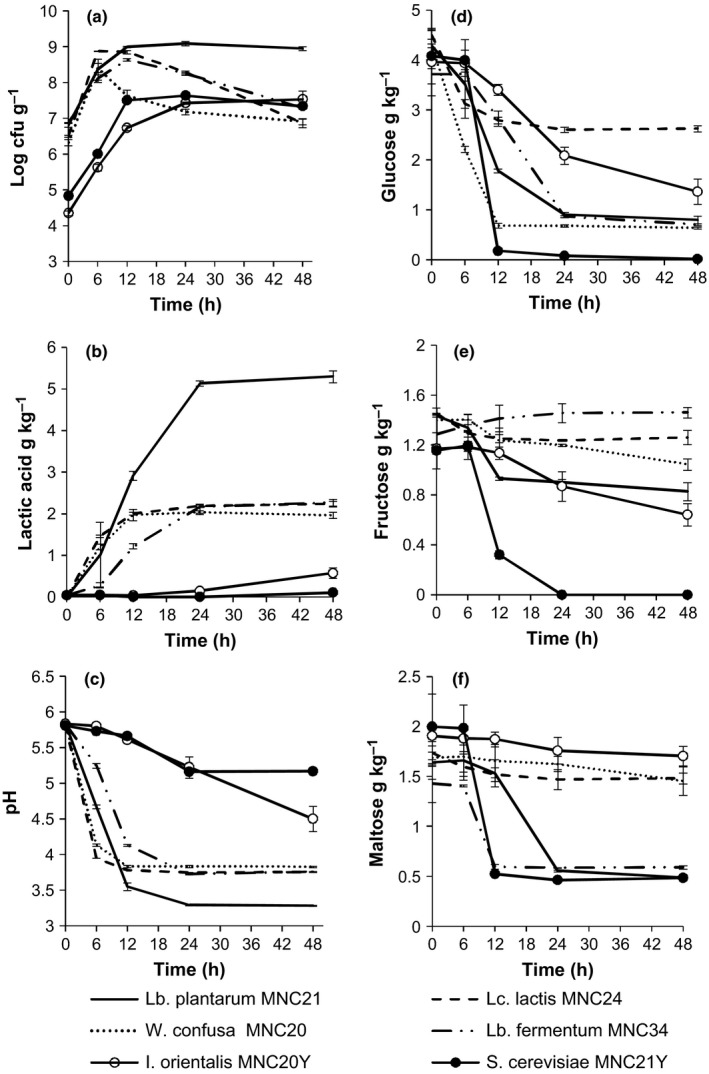
Changes in microbial counts (a), lactic acid (b), pH (c) and sugars: glucose (d), fructose (e), and maltose (f) during fermentation of *Obushera* inoculated with single lactic acid bacteria (LAB) and yeast starter cultures. Error bars show standard deviations of three independent fermentations

The study evaluated the ability of the individual LAB and yeast starter cultures to grow in *Obushera* to levels that have been reported in previous studies on naturally and starter culture fermented cereal beverages. Individual LAB and yeast starter cultures grew in *Obushera* increasing in numbers from 6.6 to 8.4–9.4 log cfu g^−1^ and 4.5 to 7.6 log cfu g^−1^, respectively within 6–24 hrs (Figure 1a). In contrast LAB and yeast counts in naturally fermented *Obushera* increased from 5.0 log cfu g^−1^ to 8.9 log cfu g^−1^ after 24 hrs and 5.2 log cfu g^−1^ to 7.6 log cfu g^−1^ in 24–48 hrs, respectively (results not shown). *Lactococcus lactis* and *W. confusa* attained maximum counts the earliest (8.4–8.8 log cfu g^−1^ in 6 hrs). Counts of *Lc. lactis, W. confusa*, and *Lb. fermentum* decreased significantly (*p* < .05) by 1.3–2.0 log cfu g^−1^ between 6 and 48 hrs while those of *Lb*. plantarum were stable at 9.0 ± 0.1 log cfu g^−1^ after 12 hrs. *Issatchenkia orientalis* produced visually disagreeable surface films, a characteristic which was also observed in spontaneously fermented *Obushera* kept beyond 48 hrs.

### 3.2 Sugar utilization by LAB and yeast starter cultures

**Figure 2:**
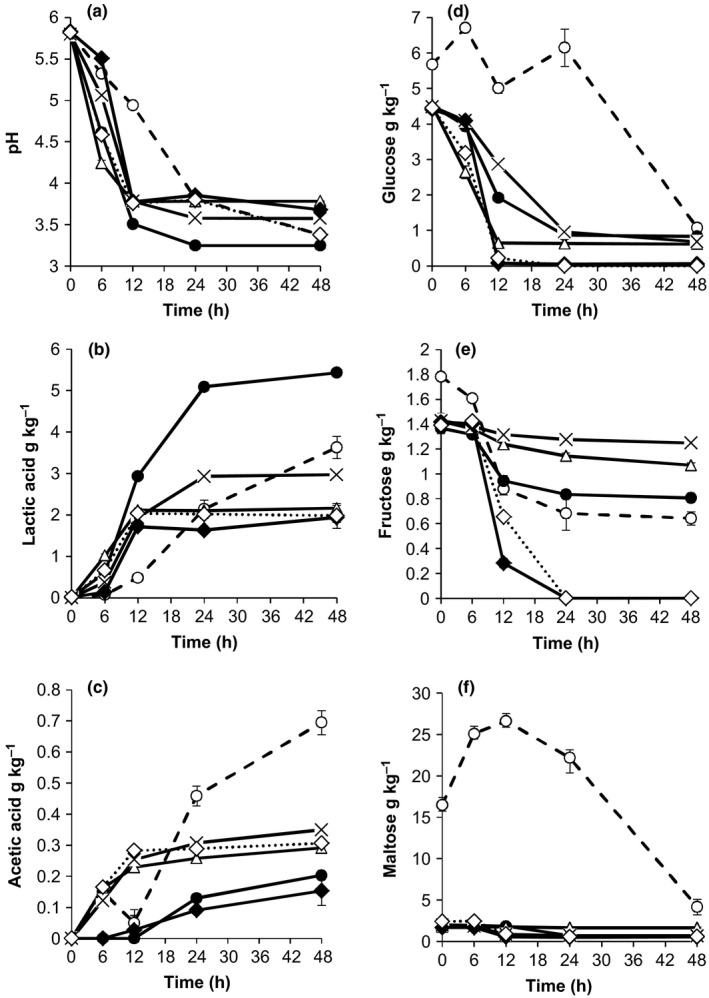
Changes in pH (a), lactate (b), acetate (c), glucose (d), fructose (e), and maltose (f) during fermentation of *Obushera* inoculated with sorghum malt (spontaneous fermentation) (ο) or mixed starter cultures: *Lb. plantarum* + *Lc. lactis* (•); *Lb. plantarum* + *Lc. lactis* + *W. confusa* (Δ); *Lb. plantarum* + *Lc. lactis* + *Lb. fermentum* (×); *Lb. plantarum* + *Lc. lactis* + *S. cerevisiae* (♦); *Lb. plantarum* + *Lc. lactis* + *W. confusa* + *S. cerevisiae* (⋄). Error bars show standard deviations of three independent fermentations

The main sugars observed in *Obushera* were maltose, glucose, and fructose. Spontaneously fermented samples (inoculated with 2% malt) contained significantly higher (*p* < .05) starting amounts of maltose (16.5 g kg^−1^), glucose (5.7 g kg^−1^) and fructose (1.8 g kg^−1^) (Figure 2d, e and f) than starter culture fermented samples (1.8 g kg^−1^ of maltose, 4.3 g kg^−1^ of glucose, and 1.4 g kg^−1^ of fructose) (Figure 1d, e and f; Figure 2d, e and f). All single starter cultures utilized glucose while *Lb. plantarum, Lb. fermentum*, and *S. cerevisiae* in addition simultaneously catabolized maltose (Figure 1d, e and f). *Lactobacillus fermentum, Lc. lactis*, and *I. orientalis* did not utilize fructose during fermentation. *Lactococcus lactis* and *W. confusa* metabolized glucose significantly (*p* < .05) faster than the other starter cultures during the first 6 hrs. *Saccharomyces cerevisiae* had the highest rate (*p* < .05) of breakdown of glucose, maltose, and fructose (reduced these to a minimum in 12–24 hrs).

### 3.3 Lactate and acetate production

Lactate and acetate were the main organic acids detected in *Obushera*. Lactate in spontaneously fermented *Obushera* increased to a maximum of 3.63 g kg^−1^ after 48 hrs (Figure 2b). Individual and mixed LAB starter cultures produced between 1.72 and 5.30 g kg^−1^ lactic acid after 12–48 hrs and in effect decreased pH from 5.8 to 3.55–3.83 within 12 hrs. However, *Lb. fermentum* monocultures and spontaneous fermentation took 24 hrs to attain similar pH values (Figure 1c and 2a). As for the yeasts, only *I. orientalis* produced a significant (*p* < .05) amount of lactate (0.57 g kg^−1^) after 48 hrs and thus reduced pH to 4.5 ± 0.2 (Figure 1b and c). Lactic acid production by *Lb. plantarum* was significantly (*p* < .05) higher than for *Lb. fermentum* and *W. confusa* and *Lc. lactis* (Figure 2b). However, monocultures of *W. confusa* and *Lc. lactis* showed significantly faster (*p* < .05) lactate production during the first 6 hrs and in effect lowered the pH of *Obushera* from 5.83 to 3.95–4.12 (Figure 1b and c). Acetate in spontaneously fermented *Obushera* increased to a maximum of 0.70** **g kg^−1^ after 48 hrs (Figure 2c). *Lactobacillus fermentum* and *W. confusa* individually, or in cocultures produced significantly (*p* < .05) higher amounts of acetic acid (0.25–0.35 g kg^−1^) than the rest of the starter cultures (≈ 0.1 g kg^−1^) (Figure 2). The *Lb. plantarum* and *Lb. plantarum* + *Lc. lactis* cultures produced the highest amounts of lactate (*p* < .05) (Figure 1 and 2). However, co‐culturing the *Lb. plantarum* + *Lc. lactis* starter culture with *S. cerevisiae, W. confusa* or *Lb. fermentum* significantly (*p* < .05) reduced lactate yields from about 5 g kg^−1^ to 2 g kg^−1^(Figure 2b).

### 3.4 Production of ethanol

Ethanol was the major alcohol produced in spontaneously fermented samples and ranged from 0.62 g kg^−1^ to 2.00 g kg^−1^ between 12 and 48 hrs (Table 2 and Figure 4). Only monocultures of *Lb. fermentum*,* W. confusa*,* I. orientalis* and *S. cerevisiae* produced ethanol with the later producing the highest (*p* < .05) amounts (1.55 g kg^−1^) compared to the rest (0.79–0.88 g kg^−1^) (Table 2). Starter cultures with *S. cerevisiae* showed the highest rate of ethanol production reaching peak concentrations (1.4–1.56 g kg^−1^) after 12 hrs (Figure 4). Ethanol production by *S. cerevisiae* was not affected by co‐culturing the yeast with LAB.

### 3.5 Production of acetaldehyde

**Figure 3:**
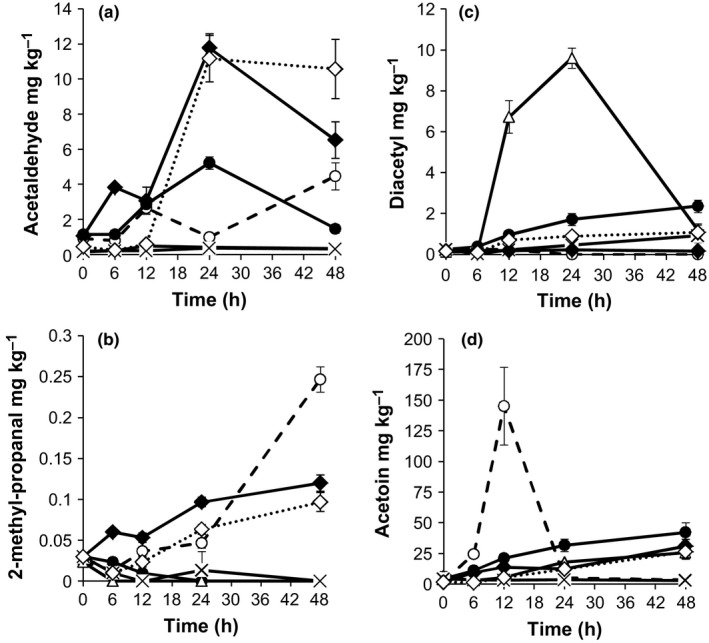
Changes in concentrations of acetaldehyde (a), 2‐methyl‐1‐propanal (b), diacetyl (c), and acetoin (d) during fermentation of *Obushera* inoculated with sorghum malt (spontaneous fermentation) (ο) or starter cultures: *Lb. plantarum* + *Lc. lactis* (•); *Lb. plantarum* + *Lc. lactis* + *W. confusa* (Δ); *Lb. plantarum* + *Lc. lactis* + *Lb. fermentum* (×); *Lb. plantarum* + *Lc. lactis* + *S. cerevisiae* (♦); *Lb. plantarum* + *Lc. lactis* + *W. confusa* + *S. cerevisiae* (⋄). Error bars show standard deviations of three independent fermentations

Acetaldehyde reached a maximum concentration of 4.46 mg kg^−1^ in spontaneously fermented samples (Figure 3a). *Issatchenkia orientalis* and *S. cerevisiae* produced the highest (*p* < .05) amounts of acetaldehyde (8.99–10.69 mg kg^−1^) followed by *Lb. plantarum* (2.14 mg kg^−1^) and *Lc. lactis* (1.76 mg kg^−1^) (Table 1).

**Table 1:** Changes in concentrations of aldehydes and ketones resulting from single starter culture and spontaneous fermentation of *Obushera,* a sorghum malt beverage after 24 hrs

Starter culture	Aldehydes(mg kg^−1^)				Ketones(mg kg^−1^)		
	Acetald	2‐ME‐pro‐al	2‐ME‐but‐al	3‐ME‐but‐al	Acetone	Diacetyl	Acetoin
Un‐inoculated (T = 0 h)	0.67 ± 0.42^a^	0.03 ± 0.02^a^	0.04 ± 0.01^a^	0.06 ± 0.03^a^	0.15 ± 0.08^a^	0.17 ± 0.06^a^	4.11 ± 2.70^a^
Spontaneous fermentation	0.98 ± 0.03b^ab^	0.05 ± 0.01^a^	0.01 ± 0.00^b^	ND	0.30 ± 0.05^ab^	ND	4.99 ± 1.64^a^
*Lb. plantarum* MNC 21	2.14 ± 0.43^b^	ND	ND	0.01 ± 0.00^a^	0.15 ± 0.02^a^	1.13 ± 0.15^b^	18.86 ± 2.15^bd^
*Lc. lactis* MNC 24	1.76 ± 0.03^b^	0.02 ± 0.00^a^	0.01 ± 0.00^b^	0.02 ± 0.0^a^	0.13 ± 0.01^a^	2.10 ± 0.21^b^	30.00 ± 6.25^c^
*Lb. fermentum* MNC 34	0.30 ± 0.01^a^	ND	ND	ND	0.55 ± 0.06^c^	ND	ND
*W. confusa* MNC 20	0.33 ± 0.04^a^	ND	ND	0.02 ± 0.01^a^	0.51 ± 0.20^bc^	9.59 ± 1.86^c^	14.45 ± 3.33^d^
*I. orientalis* MNC 20Y	8.99 ± 2.20^c^	0.04 ± 0.02^a^	0.01 ± 0.00^b^	0.02 ± 0.01^a^	0.39 ± 0.02^bc^	ND	24.89 ± 5.58^bc^
*S. cerevisiae* MNC 21Y	10.69 ± 0.83^c^	0.24 ± 0.00^b^	0.10 ± 0.00^c^	0.07 ± 0.01^a^	0.62 ± 0.11^c^	0.16 ± 0.03^a^	3.53 ± 2.27^a^

Values are means ± standard deviations of three independent fermentations. Values in the same column with similar superscripts (a – e) are not significantly different (*p* > .05). ND: not detected. Acetald (acetaldehyde); 2‐ME‐pro‐al (2‐methyl‐1‐propanal); 2‐ME‐but‐al (2‐methyl‐1‐butanal); 3‐ME‐but‐al (3‐methyl‐1‐butanal).

### 3.6 Production of diacetyl, acetoin, and acetone

Spontaneously fermented *Obushera* did not accumulate diacetyl but contained the highest amounts of acetoin after 12 hrs (Figure 3d). Among the starter cultures, *W. confusa* produced the highest (*p* < .05) amounts of diacetyl (9.59 mg kg^−1^) followed by *Lc. lactis* (2.10 mg kg^−1^) and *Lb. plantarum* (1.13 mg kg^−1^) (Table 1). All single starter cultures, except *Lb. fermentum* and *S. cerevisiae*, accumulated acetoin with the highest (*p* < .05) production in *Lc. lactis* (30.00 mg kg^−1^) followed by *Lb. plantarum* ,*I. orientalis* (18.86–24.89 mg kg^−1^), and *W. confusa* (14.45 mg kg^−1^) (Table 1). Spontaneously fermented *Obushera* also accumulated acetone (0.36–0.92 mg kg^−1^) within 12–48 hrs. Only *W. confusa*,* Lb. fermentum*,* S. *cerevisiae, and *I. orientalis* produced significant (*p* < .05) amounts (0.22–0.62 mg kg^−1^) of acetone (Table 1). *Saccharomyces cerevisiae* greatly reduced (*p* < .05) the production of diacetyl in mixed starter cultures containing *Lb. plantarum, Lc. lactis*, and/or *W. confusa* (Figure 3c).

### 3.7 Production of malty aldehydes and alcohols

**Figure 4:**
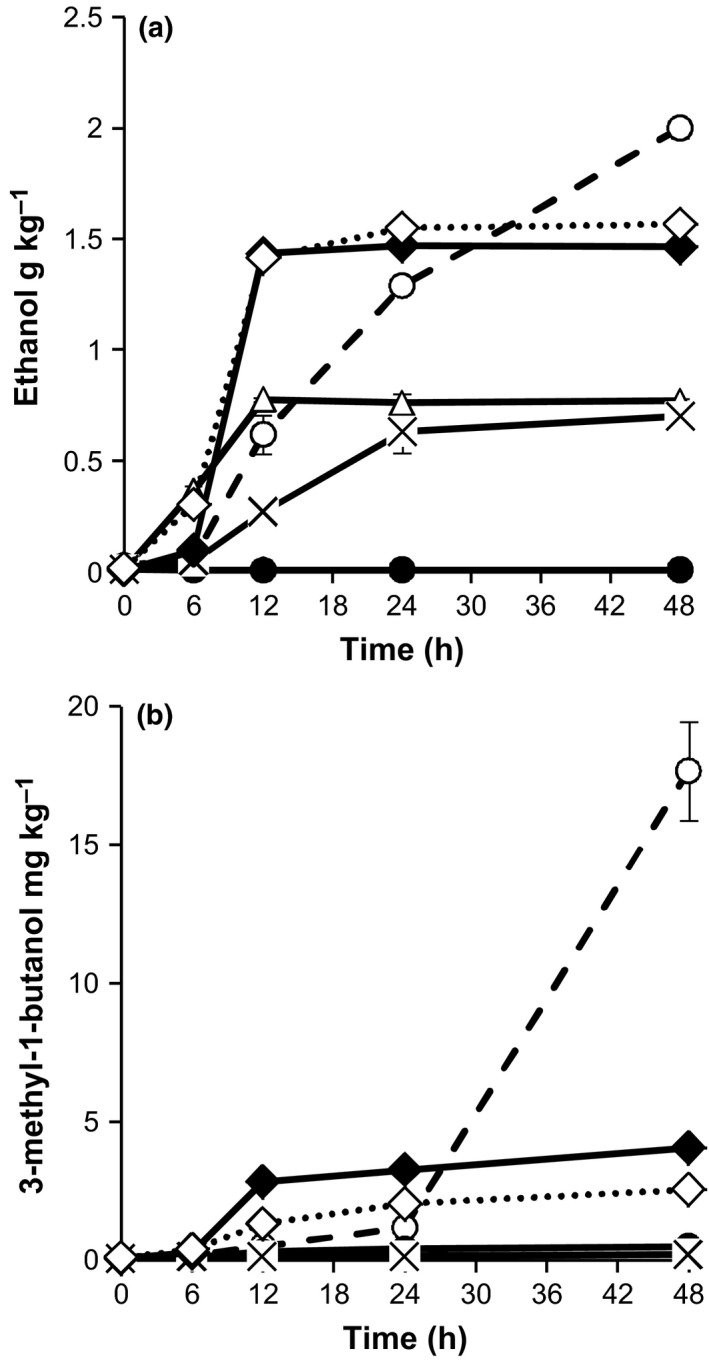
Changes in concentrations of ethanol (a) and 3‐methyl‐1‐butanol (b) during fermentation of *Obushera* inoculated with sorghum malt (spontaneous fermentation) (ο) or starter cultures: *Lb. plantarum* + *Lc. lactis* (•); *Lb. plantarum* + *Lc. lactis* + *W. confusa* (Δ); *Lb. plantarum* + *Lc. lactis* + *Lb. fermentum* (×); *Lb. plantarum* + *Lc. lactis* + *S. cerevisiae* (♦); *Lb. plantarum* + *Lc. lactis* + *W. confusa* + *S. cerevisiae* (⋄). Error bars show standard deviations of three independent fermentations

*Obushera* initially contained the malty aldehydes 2‐methylmethyl‐1‐propanal, 2‐methylmethyl‐1‐butanal, and 3‐methylmethyl‐1‐butanal, which were depleted by all single starter cultures except *S. cerevisiae* which produced 2‐methyl‐1‐propanal (0.24 mg kg^−1^) (Table 1). Spontaneously fermented *Obushera* accumulated 0.05–0.25 mg kg^−1^ of 2‐methyl‐1‐propanal between 24 and 48 hrs (Figure 3b). Methyl‐1‐propanal was only produced by *S. cerevisiae* (0.06–0.12 mg kg^−1^) (Table 1). The methyl alcohols 2‐methyl‐1‐propanol, 2‐methyl‐1‐butanol, and 3‐methyl‐1‐butanol accumulated during spontaneous fermentation to 10.11 mg kg^−1^, 3.67 mg kg^−1^and 17.67 mg kg^−1^, respectively after 48 hrs following a similar trend shown for 3‐methyl‐1‐butanol (Figure 4b). Only the yeasts: *I. orientalis* and *S. cerevisiae* produced 2‐methyl‐1‐propanol (2.78–5.18 mg kg^−1^), 2‐methyl‐1‐butanol (1.10 mg kg^−1^), and 3‐methyl‐1‐butanol (5.53–8.47 mg kg^−1^) in significant amounts (Table 2). *I. orientalis* produced significantly (*p* < .05) higher amounts of 2‐methyl‐1‐propanol and 3‐methyl‐1‐butanol than *S. cerevisiae* (Table 2).

**Table 2:** Changes in concentrations of alcohols, resulting from single starter culture and spontaneous fermentation of *Obushera* after 24 hrs

Starter culture	Ethanol(g kg^−1^)	2‐ME‐pro‐ol(mg kg^−1^)	2‐ME‐but‐ol(mg kg^−1^)	3‐ME‐but‐ol(mg kg^−1^)
Un‐fermented (T = 0 hr)	0.01 ± 0.00^a^	0.04 ± 0.00^a^	0.02 ± 0.01^a^	0.08 ± 0.06^a^
Spontaneous fermentation	1.29 ± 0.04^b^	1.82 ± 0.46^b^	0.25 ± 0.02^b^	1.17 ± 0.03^b^
*Lb. plantarum* MNC 21	0.01 ± 0.00^a^	0.11 ± 0.01^a^	0.07 ± 0.01^a^	0.40 ± 0.02^c^
*Lc. lactis* MNC 24	0.01 ± 0.00^a^	0.04 ± 0.00^a^	0.03 ± 0.01^a^	0.07 ± 0.01^a^
*Lb. fermentum* MNC 34	0.88 ± 0.03^c^	0.06 ± 0.01^a^	0.04 ± 0.01^a^	0.15 ± 0.01^ac^
*W. confusa* MNC 20	0.79 ± 0.08^d^	0.06 ± 0.00^a^	0.03 ± 0.01^a^	0.17 ± 0.05^ac^
*I. orientalis* MNC 20Y	0.83 ± 0.10 ^cd^	5.18 ± 0.47^c^	1.09 ± 0.17^c^	8.47 ± 0.60^d^
*S. cerevisiae* MNC 20Y	1.55 ± 0.01^e^	2.78 ± 0.05^d^	1.10 ± 0.09^c^	5.53 ± 0.01^e^

Values are means ± standard deviations of three independent fermentations. Values in the same column with similar superscripts (a – e) are not significantly different (*p* > .05). ND: not detected. 2‐ME‐pro‐ol (2‐methyl‐1‐propanol); 2‐ME‐but‐ol (2‐methyl‐1‐butanol); 3‐ME‐but‐ol (3‐methyl‐1‐butanol).

## 4 Discussion

### 4.1 Growth of starter cultures

Since not all microbial genera are of equal importance in fermentation (Holzapfel, 1997), candidate isolates for starter culture development have to be evaluated for their contribution during fermentation. Selecting appropriate starter cultures involves evaluating candidate strains for their viability and survival in the product, their ability to utilize the major carbohydrate substrates available, rate of acidification, and production of desirable flavor compounds among others (Holzapfel, 2002). It is also important to determine the effects of coculturing selected isolates on their activity such as acidification rate and production of flavor compounds.

With regard to growth of the starter cultures, individual LAB and yeast isolates used in the current study were able to grow in *Obushera* and attained maximum counts similar to those reported for spontaneously fermented *Obushera* (Muyanja, Narvhus, & Langsrud, 2004; Muyanja, Narvhus, & Langsrud, 2012). However, maximum counts for the starter cultures were attained in a much shorter time (12–24 hrs) than in spontaneous fermentation (24–48 hrs). Fast growth is desirable since it results in fast acidification and flavor development thus contributing toward shortening of processing time as well as ensuring product safety (Holzapfel, 2002). The decline in cell numbers of *W. confusa*,* Lc. lactis*, and *Lb. fermentum* during fermentation could imply that these strains are acid sensitive. The stability of *Lb. plantarum* counts can be attributed to the acid tolerance of the strain MNC 21, which has been observed in other studies (Byakika, 2015; Mukisa, 2012). Despite its viability and flavor production potential, *I. orientalis* was considered undesirable for production of fermented cereal beverages on the basis of surface film formation. Surface film formation by *I. orientalis* is commonly associated with spoilage of beverages (Stratford & James, 2003). Indeed, the development of surface films is one of the spoilage characteristics of *Obushera* that consumers may use to reject the product (Mukisa, 2012).

### 4.2 Utilization of sugars

The ability of starter cultures to metabolize sugars available in a substrate is an important aspect for starter culture selection. The major sugars in sorghum malt fermentations are maltose, glucose, and fructose. All starter cultures were able to utilize glucose thus enabling them to grow in and ferment the product. Differences were observed in the patterns and rates of utilization of maltose, glucose, and fructose by the individual LAB and yeast isolates. The ability of, and rate at which, an individual isolate metabolizes one or more sugars do influence its productivity and competiveness in a mixed starter culture (Gobbetti, 1998). Rapid utilization of sugars by *S. cerevisiae* makes it more competitive than LAB, when in coculture, resulting in diminished lactate production while ethanol yield was unaffected. Similar effects on lactate production were observed during sourdough fermentations when *S. cerevisiae* was cocultured with *Lb. plantarum* (Gobbetti, Corsetti, & Rossi, 1994) or *Lb. sanfranciscensis* (Gobbetti, 1998). In contrast, coculturing *Lb. brevis* with *S. cerevisiae* (Meignen et al., 2001) or *Lb. plantarum* with *I. orientalis* (Mugula et al., 2003) did not affect lactate production. It is possible that in the latter cases, LAB were faster than the yeasts in metabolizing the sugars while the inability of *I. orientalis* to utilize maltose, as seen from this study, could have made it less competitive. The rapid utilization of glucose by *W. confusa* and *Lc. lactis* in the first 8–12 hrs enables them to rapidly acidify the product to a pH below 4.5 in a period of about 6 hrs.

One key observation during the spontaneous fermentation of *Obushera* was that the concentration of maltose increased with maltose remaining above its taste threshold concentration of 13 g kg^−1^ for at least 24 hrs (Laska, Schüll, & Scheuber, 1999). This increase in sugars results from hydrolysis of starch by cereal amylases and is responsible for the desirable sweet taste of products such as *Obushera*. Therefore, low initial amounts of sugars in starter culture fermented samples, coupled by rapid depletion by the starter cultures could compromise the taste acceptability of some products for which sweetness is an important attribute. It may therefore be necessary to introduce a saccharification step prior to inoculation with starter culture in order to achieve desirable levels of sweetness in such products.

### 4.3 Lactate and acetate production

Acid production is important for both taste and safety of fermented products. Lactate imparts a sour taste which is an important sensory attribute of LAB fermented products (Mukisa et al., 2010). To ensure safety of lactic fermented products, some studies recommend attaining a rapid pH drop to ≤4.5–4.0 and a titratable acidity of about 0.7% lactic acid (Steinkraus, 1996). Fast acidification to pH below 4.0 inhibits spoilage bacteria, enteropathogens, and *Bacillus* sp (Holzapfel, 1997; Steinkraus, 1996). The ability to achieve rapid acidification in a shorter time than observed in the traditional process should therefore be one of the important criteria for selecting LAB starter cultures. All the LAB starter cultures used in this study were able to drop the pH of *Obushera* to below 4.5 in 6–12, but were not able to raise the acidity to 0.7%. Accelerated acidification by LAB starter cultures has also been reported in related studies (Mugula et al., 2003; Muyanja, Narvhus, & Langsrud, 2004; Muyanja, Narvhus, & Langsrud, 2012). Spontaneously fermented *Obushera* attained a pH below 4.5 in a much longer time (24 hrs) and only attained acidity of about 0.35%. *Lactobacillus fermentum*,* Lc. lactis*, and *W. confusa* only reached a maximum acidity of 0.2% in 12–24 hrs while *Lb. plantarum* was able to raise the acidity to 0.53% in 24 hrs. In this respect, *Lb. plantarum* would be a better candidate as a starter culture for such fermentations because of its superior acid production. This starter culture has been reported to produce up to 1.7% lactic acid from sorghum malt extracts (Byakika, 2015). High lactate production in *Lb*. *plantarum* strains is attributed to versatility in metabolism of carbohydrates coupled with tolerance to acid conditions (Van der Meulen et al., 2007). In some instances, excessive lactate production by *Lb. plantarum* could result in an undesirable sour taste (Hansen & Hansen, 1996). Therefore, the reduction in lactate production observed when *S. cerevisiae* was cocultured with the LAB‐mixed starter cultures can help prevent excessive souring during fermentation, as long as the desired level of acidity is attained. This interaction is reported to have moderated the souring effect of *Lb. plantarum* in sourdough bread (Hansen & Hansen, 1996).

Spontaneously fermented *Obushera* contained acetic acid (0.1–0.7 g kg^−1^) in amounts close to those observed previously (Muyanja, Narvhus, Treimo, & Langsrud, 2003). Levels exceeding 0.4–1.5 g L^−1^ are undesirable in wines because they impart vinegar taints (Bartowsky, Xia, Gibson, Fleet, and Henschke (2003). Excessive acetate production is also responsible for the pungent attribute which reduces the acceptability of *Obushera* (Mukisa et al., 2010). Acetic acid in concentrations of 0.1–0.2 g kg^−1^ acts as a flavor enhancer in sourdough bread (Hansen & Hansen, 1996). It is possible that the relatively low levels of acetate (0.25–0.35 g kg^−1^) produced by the starter cultures in this study might also contribute positively by enhancing the flavor of *Obushera*. Acetate production in moderate amounts could therefore be of significance in flavor of *Obushera* and related fermented cereal products thus necessitating the inclusion of heterofermentative starter culturers such as *W. confusa* and *Lb. fermentum*.

### 4.4 Ethanol production

Ethanol production in *Obushera* is responsible for the characteristic alcoholic flavor (Mukisa et al., 2010). *Obushera* of the *Obutoko* type is acceptable with an alcohol content of up to 10 g kg^−1^, but levels up to 45 g kg^−1^ result in a decline in acceptability (Mukisa, 2012). Concentrations of ethanol in sorghum based fermentations such as *Obushera* and *Togwa* vary with type of product and duration of fermentation ranging from 0.1 g kg^−1^ to 60.0 g kg^−1^ (Mugula et al., 2003; Mukisa, Muyanja, et al., 2012; Muyanja, Narvhus, & Langsrud, 2012). Ethanol in spontaneously fermented *Obushera* (1. 29–2.00 g kg^−1^ between 24 and 48 hrs) was within expected limits which do not negatively impact on acceptability*. Saccharomyces cerevisiae* produced the highest amounts of ethanol in 12 hrs (1.55 g kg^−1^), while ethanol production by *Lb. fermentum*,* I. orientalis*, and *W. confusa* was comparable (0.79–0.88 g kg^−1^). Ethanol is a product of carbohydrate metabolism by obligate heterofermentative LAB and yeasts, but the latter are generally recognized as high producers (Hansen & Schieberle, 2005). Although starter cultures had significantly lower yields of ethanol compared to spontaneously fermented *Obushera*, maximum amounts were achieved in a shorter time (12 hrs). The low yield of ethanol in samples fermented by *S. cerevisiae* can be attributed to substrate limitation while productivity of *Lb*.* fermentum* and *W. confusa* was more likely affected by increasing acidity. *Lactobacillus fermentum* was also less competitive in coculture with *Lc. lactis* and *Lb. plantarum* since these two utilized sugars much faster. It is therefore possible to use heterofermentative LAB, or yeast to attain either low or relatively high levels of ethanol in fermented cereal fermentations depending on the desired sensory attributes.

### 4.5 Production of acetaldehyde

Variations in acetaldehyde concentrations during fermentation followed a similar pattern previously observed by Muyanja, Narvhus, & Langsrud, (2012). In the current study, yeasts produced the highest concentrations of acetaldehyde (8.99–10.69 mg kg^−1^) during the first 24 hrs and were followed by *Lb. plantarum* and *Lc. lactis* (1.76–2.14 mg kg). Acetaldehyde is produced by LAB and yeasts, mainly as an intermediate in ethanol production (Liu & Pilone, 2000). However, yeasts and in particular *S. cerevisiae*, are known to produce higher amounts compared to LAB (Liu & Pilone, 2000). Coculturing *Lb. fermentum* or *W. confusa* with the *Lb. plantarum* + *Lc. lactis* starter culture prevented accumulation of acetaldehyde. The acetaldehyde was probably reduced to acetate/ethanol by action of acetaldehyde/ethanol dehydrogenase from *W. confusa* or *Lb. fermentum* (Zaunmüller, Eichert, Richter, & Unden, 2006). Acetaldehyde imparts a pleasant fruity aroma when present in low concentrations, but results in pungent odors at high concentrations (Liu & Pilone, 2000). A fruity aroma is one of the desirable sensory attributes of *Obushera* (Mukisa et al., 2010). The aroma threshold of acetaldehyde ranges from 0.015 mg kg^−1^ in water to 125 mg kg^−1^ in wine (Imhof, Glättli, & Bosset, 1994; Liu & Pilone, 2000). Amounts of acetaldehyde in naturally fermented *Obushera* fall within this range, suggesting that it is an important flavor compound. This may necessitate selection of starter cultures with the ability to accumulate acetaldehyde. In this study, the inclusion of *S. cerevisiae* in the LAB starter culture or elimination of *W. confusa* or *Lb. fermentum* from the *Lb. plantarum* + *Lc. lactis* ensured accumulation of acetaldehyde.

### 4.6 Production of diacetyl, acetoin, and acetone

Diacetyl and acetoin have been previously detected in *Obushera* in ranges of 0.2–2.4 mg kg^−1^ and 1.3–41.6 mg kg^−1^, respectively (Muyanja et al., 2003). However, in this study only acetoin was detected. Diacetyl and acetoin are intermediate products of reductive decarboxlaytion of excess pyruvate to 2,3‐butanediol (Bartowsky & Pretorius, 2009). The pyruvate is obtained from sugar or citrate metabolism. Diacetyl is commonly produced by citrate positive *Lactococci*,* Lactobacilli*, and yeasts (Bartowsky & Pretorius, 2009). Yeasts further rapidly convert diacetyl to acetoin and finally 2,3 butanediol (Bartowsky & Pretorius, 2009). In this study, *W. confusa* produced the highest amounts of diacetyl, but these were reduced in coculture with *S. cerevisiae*. The diacetyl produced by *W. confusa* in a coculture was possibly rapidly converted into 2,3 butanediol by the action of *S. cerevisiae* acetoin reductase (Bartowsky & Pretorius, 2009). Diacetyl and acetoin impart buttery flavors, but diacetyl is more important owing to its lower flavor threshold (0.002–2.2 mg L^−1^) compared to that of acetoin (150 mg L^−1^) (Bartowsky & Pretorius, 2009; Imhof et al., 1994). Diacetyl concentrations above 5–7 mg L^−1^ impart undesirable flavors in wines (Bartowsky & Pretorius, 2009). Use of *W. confusa* alone or in combination with *Lc. lactis* and *Lb. plantarum* could result in excess production of diacetyl and hence impart undesirable flavor. However, this can be minimized by coculturing with *S. cerevisiae*.

### 4.7 Production of malty aldehydes and alcohols

The aldehydes 2‐methyl‐1‐propanal, 2‐methyl‐1‐butanal, and 3‐methyl‐1‐butanal are associated with malty flavors (Sheldon, Lindsay, Libbey, & Morgan, 1971) while their corresponding alcohols, at concentrations below 300 mg kg^−1^, impart desirable fruity notes to wine (Bartowsky & Pretorius, 2009). The higher alcohols could also be responsible for the desirable fruity notes in *Obushera* (Mukisa et al., 2010). The taste threshold concentrations of 2‐methyl‐1‐propanal, 2‐methyl‐1‐butanal, and 3‐methyl‐1‐butanal are 0.18, 0.13, and 0.06 mg kg^−1^, while those of their corresponding alcohols are 5.25, 5.50, and 4.75 mg kg^−1^ (Sheldon et al., 1971). These compounds have been detected in *Obushera* in amounts close to or above their thresholds indicating their importance in *Obushera* flavor (Muyanja, Narvhus, & Langsrud, 2012). Malty compounds are produced via transamination of specific amino acids to α‐keto acids followed by decarboxylation into the aldehyde and reduction to the corresponding alcohol (Hansen & Schieberle, 2005; Hazelwood, Daran, van Maris, Pronk, & Dickinson, 2008). Catabolism of valine, isoleucine, and leucine yields the aldehydes 2‐methyl‐1‐propanal, 2‐methyl‐1‐butanal, and 3‐methyl‐1‐butanal, respectively (Hazelwood et al., 2008). Their production is reported in LAB and yeasts although yeasts are often regarded as the main producers (Gobbetti, 1998; Hazelwood et al., 2008). This study indicates that yeasts are the major producers of malty compounds in sorghum malt fermentation. Thus, the inclusion of yeasts as part of the starter culture in some cereal fermented products might be inevitable. In this study, *Obushera* fermented by the *S. cerevisiae* starter culture yielded less malty compounds after 48 hrs compared to the spontaneous fermentation. This possibly resulted from a reduction in yeast metabolism following depletion of fermentable sugars, which were also initially lower in starter culture fermented samples.

## 5 Conclusion

This study reveals that besides producing lactic acid, LAB in cereal fermentations may also contribute to production of other flavor compounds notably acetate, ethanol, acetaldehyde, diacetyl, acetone, and acetoin. Yeasts mainly contribute toward production of ethanol, acetaldehyde, methyl aldehydes, and methyl alcohols. With regard to acidification, *Lb. plantarum, Lc. lactis, W*. *confusa*, and *Lb. fermentum*, or their selected combinations can be used to achieve accelerated fermentation of cereal products. However, use, or inclusion, of *Lb. plantarum* is encouraged because of its high acid production potential and stability to acid. Despite the acid sensitivity of *Lc. lactis* and *Wc. confusa*, their fast growth and glucose metabolism may contribute toward rapid acidification. Addition of *S. cerevisiae* to LAB starter cultures may be crucial for the attainment of a flavor profiles typical of traditional fermented cereal products. *S. cerevisiae* also prevents excessive lactate and diacetyl production, a phenomenon which could negatively or positively affect the flavor profile. The starter culture combinations: *Lb. plantarum* + *Lc*. *lactis* + *S. cerevisiae*, or *Lb. plantarum* + *Lc. lactis +W. confusa* + *S. cerevisiae* can be adopted for the fermentation of *Obushera*.

## Conflict of Interest

There is no conflict of interest to be declared.
